# Nonlinearity of the Upconversion Response of Er^3+^ in Y_2_TiO_5_:Er^3+^,Yb^3+^ Ceramics When Varying the Wavelength of Incident NIR Excitation Radiation

**DOI:** 10.3390/ma17163994

**Published:** 2024-08-11

**Authors:** Liviu Dudaș, Daniela Berger, Cristian Matei

**Affiliations:** Faculty of Chemical Engineering and Biotechnologies, National University of Science and Technology Politehnica Bucharest, 1-7 Gheorghe Polizu Street, 011061 Bucharest, Romania; liviu_dudas@yahoo.com (L.D.); daniela.berger@upb.ro (D.B.)

**Keywords:** Y_2_TiO_5_:Er^3+^,Yb^3+^, upconversion, sol–gel method, photoluminescence

## Abstract

The upconversion response of Er^3+^ sensitized by Yb^3+^ in various crystalline hosts and illuminated with a laser light at around 980 nm revealed certain spectral shapes that are typical for each of the crystalline matrices containing the dopants. The purpose of this work was to measure the upconversion response of Er^3+^ as a dopant in Y_2_TiO_5_, sensitized by Yb^3+^, at different concentrations relative to the substituted Y^3+^ ion, and to reveal the subtleties of the mechanisms of the energy transfers between them and the lattice. Therefore, we synthesized Y_2_TiO_5_ ceramic samples doped with different concentrations of Er^3+^ and Yb^3+^, below 10% (mol), in order to minimize the distortion of the lattice. The oxide powders, obtained using the sol–gel method, as well as the ceramics were structurally and morphologically characterized using an X-ray diffraction analysis and scanning electron microscopy. When the ceramic samples were irradiated with an NIR laser light, it was found that, at a wavelength variation of only 2 nm of the incident radiation, from 973.5 nm to 975.5 nm, the upconversion spectra differed significantly. This nonlinearity is notable because it is not present in the case of other crystalline host matrices studied by us since the literature lacks information on this subject. We also correlated this effect with the simulated distribution of the average distances between Er^3+^ and Yb^3+^ ions in the host matrix.

## 1. Introduction

Upconversion (UC) is the process by which a system, be it an atom, an ion, or a molecule, through absorbing low-energy quanta from the environment, either simultaneously or in successive steps reaches a state from which the de-excitation, either to the ground or to an intermediate level, is achieved by emitting a higher energy quanta compared with the absorbed ones. The energy absorption process is a succession of events like ground-state absorption (GSA), and one or multiple excited-state absorptions (ESAs). The absorptions of the energy quanta could be resonant, phononic-assisted, sensitized, or cooperative sensitized etc. [1].

Different nanoparticles with UC properties, designed for either high conversion efficiency or tunable color, have been studied for biomedical applications, such as biosensing and bioassays, high-contrast agents, drug delivery, and various therapies [2]. In this area, the use of IR radiation has some major advantages over its counterparts that use higher energy radiation (UV or blue light) due to a deeper penetration into biological tissue and because it is much safer.

The UC of the Er^3+^:Yb^3+^ pair, in various crystalline and biological compatible hosts, is used in medical engineering, where it acts as an in situ nanoscale, high-fidelity temperature sensor, by probing the ^2^H_11/2_,^4^S_3/2_→^4^I_15/2_ fluorescence emissions intensity ratio, which depends on the energy level populations, which in turn are influenced by temperature [3,4,5,6,7,8,9]. Also, Er^3+^:Yb^3+^ upconverting nanoparticles, due to their low energy excitation wavelength, can act as 980 nm excited triggers for drug delivery when combined with drug carrier particles [10].

In optical engineering, e.g., in fiber optics signal amplifiers, Yb^3+^ is used to enhance the Er^3+^ emission of ^4^I_13/2_→^4^I_15/2_ (1500 nm), using 980 nm as the pumping radiation, which is cheaper to obtain and has better efficiency [11,12,13,14,15].

Er^3+^:Yb^3+^ UC nanoparticles were also applied in pollutant detection and removal [16], efficiency increasing of the photocatalytic activity of TiO_2_ [17], lamp phosphors, display panels, catalysts, sensors, and photovoltaic process enhancers [18].

The main goal of the studies of the Er^3+^:Yb^3+^ activator–sensitizer pair is to increase the UC efficiency of Er^3+^ in incident near-infrared (NIR) radiation. The NIR light is easily obtained today with cheap laser diodes. Many crystals were used as hosts for this ionic pair, e.g., Y_2_O_3_, BaGd_2_ZnO_5_, and NaYF_4_ [19,20,21,22,23], each having a certain efficiency that is linked to the phononic interactions between Er^3+^ and Yb^3+^ and the crystalline lattice host.

Nevertheless, a subtlety of the mechanism of the UC process, i.e., why the red–green emission ratio of Er^3+^ ions is higher when the relative concentration of Yb^3+^ to that of Er^3+^ is increased, is not well understood. Only one paper was found addressing this issue [24], but it is not explanatory enough.

In order to better grasp the causes of this phenomenon, another crystal host, namely Y_2_TiO_5_ ceramic doped with Er^3+^ and Yb^3+^, is studied in this work. This ceramic was chosen not only because, to the best of our knowledge, the literature lacks information regarding the UC of Er^3+^ embedded in this matrix, but also because Y_2_TiO_5_ is special as the titanates display a wealth of useful characteristics like a large bandgap (≥3.3 eV [25]), thermal stability [25], photocatalytic properties, and they do not pose biological hazards [26]. Also, yttrium titanate ceramics display various phase states, which are very useful in the fission energy industry [27]. They can accommodate large defects in the crystalline matrix induced by high-intensity particle radiation or by allowing small-molecule gases (helium, hydrogen) to penetrate the lattice [28].

We selected Er^3+^ as the activator and Yb^3+^ as the sensitizer because the ^2^F_5/2_→^2^F_7/2_ transition energy of Yb^3+^ is quasi-resonant with the ^4^I_11/2_←^4^I_15/2_, ^4^F_7/2_←^4^I_11/2_ or ^4^S_3/2_←^4^I_13/2_ transitions of Er^3+^ [29], ensuring an easier energy transfer between these ions [1]. Also, the Er^3+^ energy levels are appropriately distanced between themselves [29] in order for the excited states to have a sufficiently long lifetime, such that absorption of other quanta happens before de-excitation and the UC is facilitated.

The aim of this study was to investigate how the conformation of the Er^3+^, Yb^3^ doped Y_2_TiO_5_ crystalline matrix influences the UC spectral response of Er^3+^ upon illumination at 976 nm. Hence, we compared the upconversion spectra of Er^3+^ in Y_2_TiO_5_ (YTO) with those in other host crystalline matrices, either previously studied by us or reported in the literature.

During this endeavor, we found that the UC process for this Y_2_TiO_5_ matrix is not linear and displays high sensitivity to the wavelength of the incident IR laser light, with variations as small as 2 nm leading to notable changes in the spectral composition of the upconversion emission in the visible range. This behavior is peculiar for this crystalline lattice, since Er^3+^ present in other crystalline hosts (e.g., Y_2_O_3_, BaGd_2_ZnO_5_) displays more linear characteristics, as we observed in our research.

Herein, we report for the first time this nonlinear behavior of the UC process of Er^3+^:Yb^3+^ doped ceramics, nonlinearity that is linked to the crystalline structure of Y_2_TiO_5_ both at the nanoscale and to the shape of the surrounding coordination polyhedra at the locus of Y^3+^ substitution.

This research consists also in improving the understanding of the subtleties of the energy transfer between the dopant ions and with the lattice by correlating the experimental data with simulations of the dopant distributions in the crystal and their relative interionic distances.

This nonlinearity could find different applications, such as cheap laser wavelength indicators, checking of structural modifications at the nanoscale level in materials, detecting forgeries, 980 nm laser tuning, or doppler sensors for noninertial fiber gyroscopes.

## 2. Materials and Methods

### 2.1. Materials Used and Dopant Concentrations Labelling

The synthesis of Y_2_TiO_5_ ceramics, doped with Er and Yb samples, was carried out in two steps: first, by obtaining the precursor metallic oxides using the sol–gel technique; second, these oxides were then molded into pellets using high pressure, and the pellets were sintered at a high temperature and atmospheric pressure.

The reagents used, erbium(III) nitrate pentahydrate, ytterbium(III) nitrate pentahydrate, yttrium(III) nitrate hexahydrate, titanium(IV) butoxide 97% (TB), citric acid (CA), and ethylene glycol (EG) were purchased from Sigma Aldrich (Merck Group, Darmstadt, Germany) and were used as received.

Table 1 presents the chosen concentrations of the dopant ions for replacing yttrium atoms in Y_2_TiO_5_ and the sample labeling that will be used throughout this text.

YTO 0-0 was chosen as the reference for the crystal structure when all the conditions of synthesis were the same. Also, YTO 0-0 was used to investigate the response of the pure Y_2_TiO_5_ matrix at a 976 nm illumination (fixing the background). YTO 0-1 was chosen to investigate the response of Yb^3+^ ions at the 976 nm range and observe the energy that the emitted photons have. The dopant concentration values were chosen to be small enough such that the Er^3+^ and Yb^3+^ ions should replace the Y^3+^ ions in the Y_2_TiO_5_ matrix with the lowest possible lattice distortions. As it will be seen, the lattice is very sensitive to these dopants, and concentration values higher than 10% (relative to Y^3+^) strongly hinder the equilibrium of the orthorhombic phase of Y_2_TiO_5_.

### 2.2. Synthesis of Oxide Powders

The metal nitrates in the appropriate quantities, CA (2 moles for every mol of metallic ions) and TB were dissolved in EG. The utmost care was taken for no traces of water to be in the reaction mixture since water induces the quick hydrolysis of TB. The reaction mixture was heated at 120 °C under continuous magnetic stirring (300 rpm) for slow evaporation until a transparent and thick gel was formed. The gel was thermally treated at 400 °C for 3 h until the most carbonaceous compounds were removed, and a grayish powder was obtained. The as-synthesized powders were calcinated at 900 °C for 3 h, after which their color changed from grey to pinkish due to the presence of Er^3+^ ions.

### 2.3. Obtaining of Er, Yb Doped Y_2_TiO_5_ Ceramics

Y_2_TiO_5_:Er,Yb ceramic pellets of 13 mm diameter and 1 mm thickness were obtained in two steps: first, the powders were pressed at 200 bar for 2 min, followed by grinding the resulting pellets in an agate mortar; second, the powders were pressed at 100 bar for 1 min with a slow release of the pressure. This pressing procedure was chosen to avoid the brittleness of the resulting ceramics because the initial powders had a very low cohesiveness. The first step ensures an aggregation of the particles into larger ones, whereas the second step eliminated the voids created between them, while inserting smaller inner tensions and preventing the pellet from cracking during manipulation and thermal treatment.

Two pellets for each composition were produced and placed, one on top of the other, in alumina crucibles. This stacking prevents the accidental modification (contamination, evaporation of constituents, etc.) of the composition of the touching faces of the pellets during the prolonged oven time.

The pellets were sintered at 1250 °C for 16 h. This is the minimum time for the sintering treatment for the Y_2_TiO_5_ crystal lattice stabilization. The temperature was chosen according to the reported results [30], which showed that the diffusion coefficients for oxygen ions in the Y_2_TiO_5_ structure are sufficiently high even at temperatures lower than the melting point. This ensures the formation of the orthorhombic phase while impending the segregation of phases with a lower energy of formation (−3.98 eV/atom for Y_2_O_3_ [25]) than Y_2_TiO_5_ (−3.87 eV/atom [25]) or other phases like hexagonal, pyrochlore, fluorite, or compounds like Y_2_Ti_2_O_7_ and YTiO_3_ [31].

### 2.4. Characterization of Y_2_TiO_5_ Samples

The oxide powders and the ceramics were characterized using X-ray diffraction (XRD) with a Rigaku Miniflex II, Tokyo, Japan, diffractometer with Cu Kα radiation in the 2*θ* range of 10°–70°, at a 2 °/min rate, and a step of 0.01°. The scanning electron microscopy (SEM) was performed using a Tescan Vega 3LM, Brno, Czech Republic, microscope equipped with an EDS spectrometer. The upconversion spectra were measured using a USB4000CG-UV-NIR spectrometer from Ocean Optics (Orlando, FL, USA) and the OceanView software version 1.6.7. The LCU98E042Ap diode (Laser Components GmbH, Olching, Germany) was driven with emitted power control and thermally stabilized because the lasing wavelength varies with temperature. The thermal control allowed for the IR laser wavelength tunability with subnanometer precision. The ray was collimated with a standard laser collimator, which illuminated an area of 0.5 mm^2^, allowing for individual microcrystal probing. The acquisition fiber optic head entrance was at a distance of 3 mm from the illuminated spot and had a diameter of 200 µm.

The spectra acquisition periods for the photon counting were 30 ms, and the laser illumination power was 150 mW. All the measurements were averaged over 10 full-spectrum acquisitions, such that a single averaged spectrum was measured in 0.3 s.

These power and time parameters were chosen so as not to saturate the spectrometer sensor in the spectral range of interest (visible). The measurement setup for the upconversion spectra is depicted in Figure S1 (Supplementary Materials).

## 3. Results

### 3.1. Powder Oxides XRD Characterization

**Figure 1:**
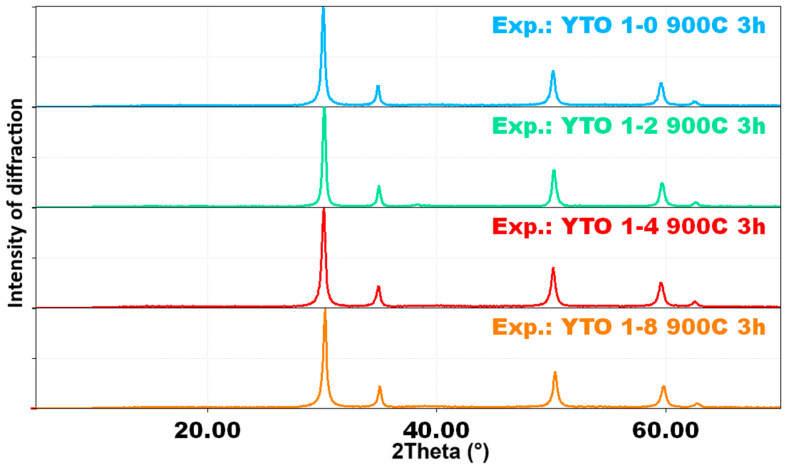
XRD patterns of the YTO 1-0, YTO 1-2, YTO 1-4, and YTO 1-8 oxidic powders obtained at 900 °C for 3 h. Observe the patterns typical of a cubic phase, which is the precursor of the orthorhombic Y_2_TiO_5_.

The XRD analysis of the resulting oxide powders reveals smooth and wide peaks, indicating the formation of a cubic phase, which is the precursor of the orthorhombic Y_2_TiO_5_ (formed after the sintering), and is presented in Figure 1.

### 3.2. XRD Analysis of Y_2_TiO_5_:Er,Yb Ceramics

**Figure 2:**
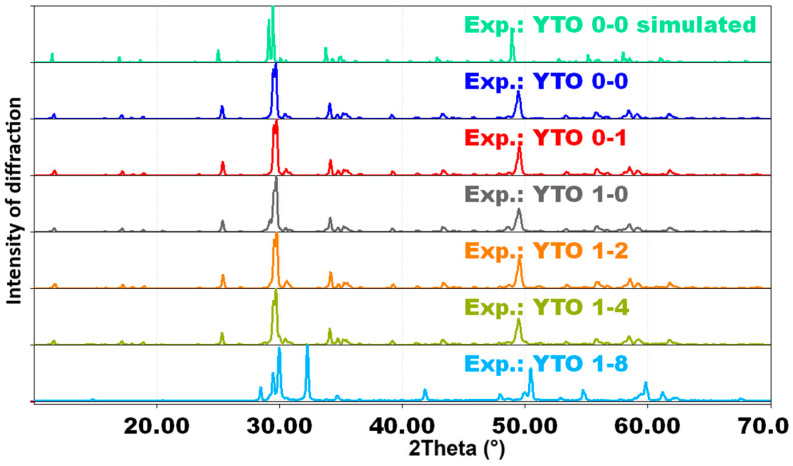
XRD patterns for simulated orthorhombic Y_2_TiO_5_ and measured for the YTO 0-0, YTO 0-1, YTO 1-0, YTO 1-2, YTO 1-4, and YTO 1-8 ceramics obtained at 1250 °C. For the other ceramics, see also Figure S2. In the case of YTO 1-8, the distortion from orthorhombic Y_2_TiO_5_ is stark.

The XRD patterns of the YTO 0-0, 0-1, 1-0, 1-2, 1-4, and 1-8 ceramics, as well as the simulated orthorhombic YTO 0-0 diffractogram using the VESTA 3 software [32], can be seen in Figure 2. The XRD patterns of the other Y_2_TiO_5_:Er,Yb samples are presented in Figure S2 (Supplementary Materials). It was found that the sintering process at 1250 °C for 16 h is the optimum thermal treatment to obtain the orthorhombic Y_2_TiO_5_ phase. Other attempts, i.e., a lower calcination temperature or shorter time, resulted in a mixture of cubic and orthorhombic phases, whereas higher temperatures gave rise to other phases segregation.

In the case of YTO 1-4, the matrix begins to be distorted (a small peak at 2*θ* = 30° can be observed), and for YTO 1-8, the distortions are even higher. These deformations are compatible with the undoped structure because of the large interstitial (no bond) spaces and the fact that Yb^3+^ ions have almost a 4% smaller ionic radii than those of Y^3+^ ions.

### 3.3. Crystallinity of the Ceramic Pellets

**Figure 3:**
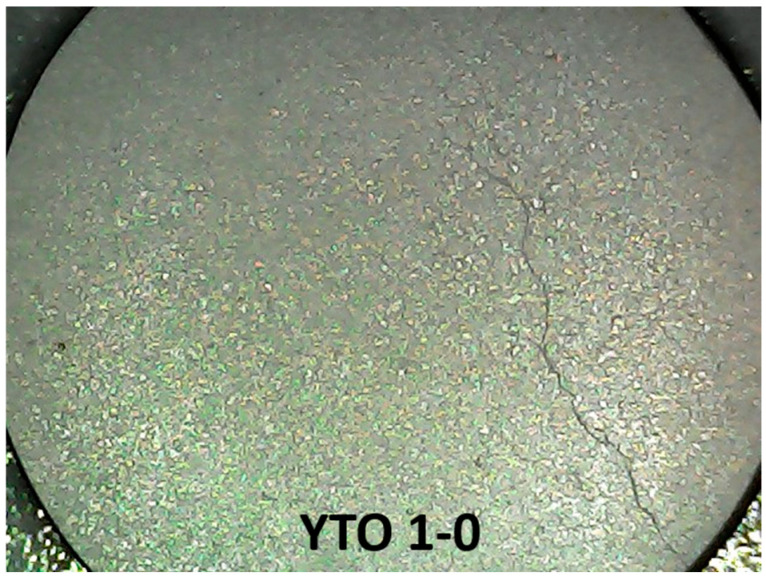
Image of a ceramic pellet surface for YTO 1-0 after polishing. The illumination is frontal from the LED lights of the microscope. The surface resembles a mosaic made by uniform-sized shiny microcrystals with slightly different orientations. The crack is an indication of the brittleness of the ceramic due to the small cohesiveness of the oxide particles.

After sintering, the protected face of each ceramic pellet was first sanded with 2000 grit, then polished with 6000 grit until the face was gloss-shining. The procedure revealed, only under frontal illumination with a bright white light, the microcrystal structures of the pellets. An example of the polished face of YTO 1-0 is shown in Figure 3 (see also Figure S3), and one can see that the sizes of the microstructures are uniform.

The uniformity of the microcrystal size for each dopant concentration shows a successful sintering process. The variation in the average size of the microcrystals shows how the dopant concentration influences the crystallization process. There is a variation in the microstructure sizes across the cases of dopant concentrations, with the lowest feature sizes being for YTO 1-0 (Figure 3) and the largest ones for YTO 1-2 (Figure S3). This is an indication that the doping ions (even if the total dopant concentration relative to Y is below 10%) strongly influence the crystallization process and the crystalline phase formation (Table S1) revealed by the XRD patterns (Figure 2).

### 3.4. Structure of the Y_2_TiO_5_:Er,Yb Crystal Unit Cell

**Figure 4:**
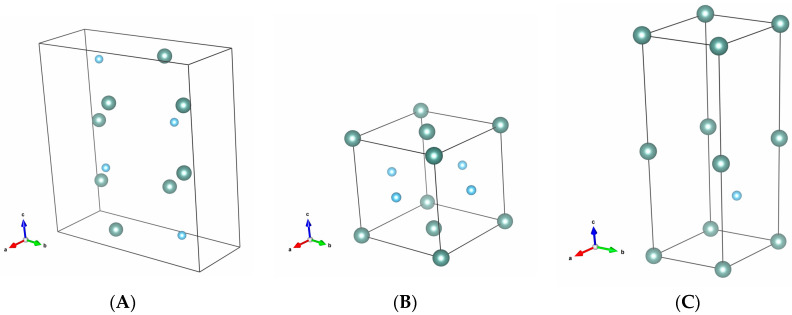
(**A**) Orthorhombic unit cell of Y_2_TiO_5_; (**B**) Fluorite unit cell of Y_2_Ti_2_O_7_ with edges of 5.15 Å found in pellets with high Yb^3+^ concentrations of 6% and 8%; (**C**) hexagonal unit cell of β-Y_2_TiO_5_ with edges *a* = 3.615 Å, *c* = 11.384 Å [31]; (only the cations shown: Yb^3+^ larger grey spheres, Ti^4+^ smaller light blue spheres). Notice, in the case of orthorhombic phase, the large distances between Y^3+^ ions.

Y_2_TiO_5_ crystallizes in the orthorhombic system with P1 symmetry. The unit cell parameters are: *a* = 3.72 Å, *b* = 10.45 Å, and *c* = 11.35 Å. The CIF data [25] regarding the Y_2_TiO_5_ compound were visualized using the VESTA 3 software [32] and reveal the positions of the ions in the unit cell, as depicted in Figure 4.

Yttrium ions are coordinated by seven oxygen ions, and Ti^4+^ ions are coordinated by five oxygen ions. All Y^3+^ ions have the same geometry of the coordination polyhedra.

**Figure 5:**
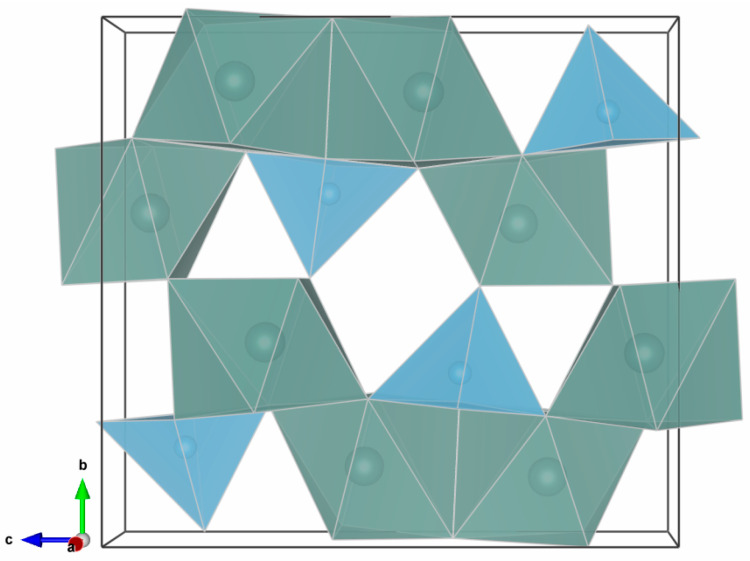
Polyhedral view of the unit cell of the orthorhombic crystal structure of Y_2_TiO_5_. Y^3+^ coordination polyhedra with green–gray, Ti^4+^ coordination polyhedra with light blue. The unit cell has large interstitial spaces, which are the main cause of the phase instability when the dopant concentrations exceed certain threshold values.

It can be observed (Figure 5 and Figure S4) that the orthorhombic matrix has large voids, which can easily give rise to lattice defects, nanostructural instability, the accommodation of other kinds of ions, or being permeable to gases with low atomic radii. The ionic radii for the involved metallic ions, coordinated by seven oxygen ions, are provided in Table 2. Yb^3+^ has a 4% smaller radius compared with Y^3+^ [33]. Therefore, larger concentrations of Yb^3+^ hinder the shape and stability of the orthorhombic Y_2_TiO_5_.

The XRD phase matching analysis of the samples performed using the Match! software [34] shows that, for each ceramic, there is a combination of phases that are compatible with the orthorhombic structure of Y_2_TiO_5_ (Table S1). This is due to the interstitial spaces of the structure, as seen in Figure 4 and Figure 5, which allow for such a variability of combinations of phases with the same structure of the unit cell but with slightly different geometrical parameters; the percentage of the combinations is also influenced by the dopant concentrations. In the case of YTO 1-8 and YTO 3-6, which have high dopant concentrations, fluorite Y_2_Ti_2_O_7_ and even hexagonal Y_2_TiO_5_ were formed.

Consulting the phase diagram for Y_2_O_3_–Y_2_Ti_2_O_7_ [31], one can conclude that the phase composition is very sensitive to the types of doping ions and their concentrations. Even low concentrations of doping ions can significantly alter the phase equilibrium, and this is due to the metastable conformation of the matrix.

In the cases of YTO 1-8 and YTO 3-6 with a high concentration of Yb^3+^, the XRD analysis showed (Figure 2 and Figure S2), beside the expected orthorhombic phase, a hexagonal phase and the Y_2_Ti_2_O_7_ fluorite phase. The YTO 1-8 ceramic was obtained also trying slightly different sintering conditions (at a maximum temperature of 1300 °C), but the hexagonal and fluorite phases appeared in all cases, which is an indication that Yb^3+^ promoted the formation of the Y_2_Ti_2_O_7_ phase, supposedly because of its lower ionic radius (0.925 Å) than that of the Y^3+^ radius (0.960 Å).

### 3.5. SEM Investigation of the Sintered Powders and Ceramics

**Figure 6:**
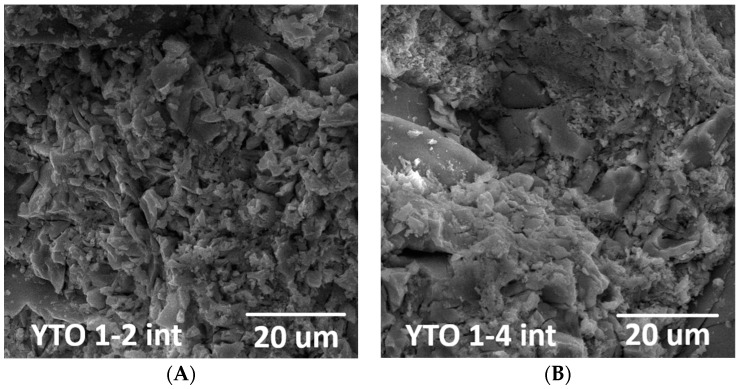
SEM images for ceramic pellet transversal sections (magnification: ×2000) (**A**) YTO 1-2, (**B**) YTO 1-4. For all the other cases, including SEM images for calcined powders, see Figure S5 (Supplementary Materials). SEM images of ceramic samples show that, during the sintering process, oxide particles did not aggregate, keeping the crystallinity of Y_2_TiO_5_ at a short range level.

The SEM images show that the powders (calcined also at 1250 °C) and the ceramics have very similar aspects, with the pellets being more compact (Figure 6 and Figure S5). Comparing with other morphologies shows that the closest matching one is that of volcanic ash [35,36].

In all cases, there is no evidence of any forms of large-scale crystals, but only a mix of irregular shards with a large range of dimensions, which is an indication that the good crystal structure, revealed using XRD, is only at the nanoscale level and that these nanocrystals are attached among themselves in a macroscopic phase.

This aspect is allegedly due to the loose structure of the YTO crystal unit cell, which allows for deformations and other inter-nanocrystalline transition phases, which could also be related to the variate compositions revealed using the X-ray diffractograms (Table S1). Also, it can be observed that the higher the total dopant concentration, the smaller the ceramic grains, while the aspect of the powders remains the same (Figure S5).

### 3.6. Upconversion Light on a Wide Area

**Figure 7:**
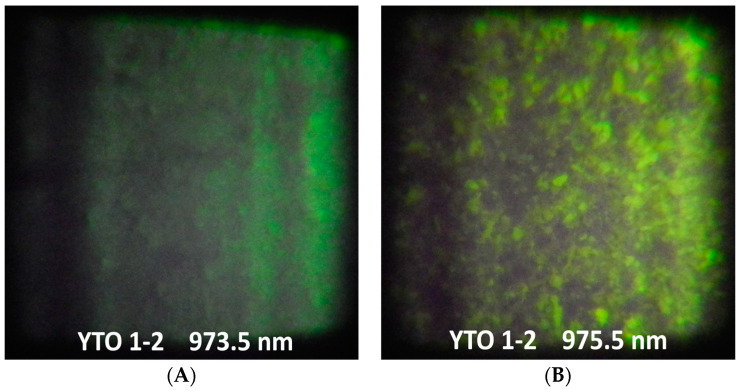
The emissions of the pellets at small angle (from right) illumination with 973.5 nm (**A**) and 975.5 nm (**B**) radiation for YTO 1-2. One can observe the spots and the differences in the UC response between the illumination cases. Larger crystals shine brighter than the smaller ones revealing nanodot-like energy confinement. The vertical shadows are due to the nonuniformity of the laser field of the diode.

Prior to the upconversion emission spectroscopic investigation, the pellets were tested by illuminating them with a grazing IR laser light with two wavelengths, 973.5 nm and 975.5 nm, over an area of about 3 mm × 3.5 mm with a power of 175 mW. The pictures are presented in Figure 7 for YTO 1-2 (for the other cases, see Figure S6). The microcrystals shine the upconverted radiation differently, revealing a mosaic-like local variation in brightness (best seen in the YTO 1-2 case, Figure 7A,B) and bright spots in the other cases (Figure S6).

This brightness variability is due to the size of the crystals and not caused by dopant concentration nonuniformity, because the EDX analysis (Figure S7) revealed a homogeneous distribution of the dopants. This is notable, especially in the cases with higher concentrations of Er^3+^, YTO 2-4, YTO 4-4, and YTO 3-6, with spots having slightly green or red hues, yet the spectral shapes being the same. The stripe variation in brightness is due to the unevenness of the laser field emitted by the diode.

How the dimension of the microcrystal influences the upconversion mechanism remains to be established, but the images strongly suggest that the sizes of the microcrystals are influencing the phononic energy transfers and/or nanodot-like energy confinement.

Reorienting the pellets relative to the polarization of the laser light shows no variability in the brightness of the spots, underscoring the idea that the energy transfers are isotropic, not depending on some preferred light–crystal relative orientations.

### 3.7. Upconversion Spectra

The specifics of the UC spectra measurement we used (pinpoint focusing the laser light) allowed us to probe the individual spots on the pellets and check the variability of the response spectra across their surface.

**Figure 8:**
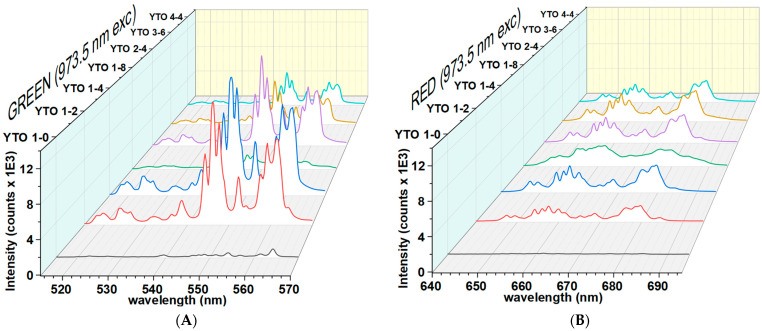
Visible upconversion spectra at a 973.5 nm illumination. (**A**): emissions from the ^4^H_11/2_,^4^S_3/2_→^4^I_15/2_ (green) transitions and (**B**): emissions from the ^4^F_9/2_→^4^I_15/2_ (red) transitions. The green emissions are brighter than the red ones, but in the case of YTO 1-8, the green emission is suppressed, showing that large Yb^3+^ concentrations not only distort the orthorhombic phase of Y_2_TiO_5_, but hinder the populating and/or stability of ^4^S_3/2_ level of Er^3+^.

**Figure 9:**
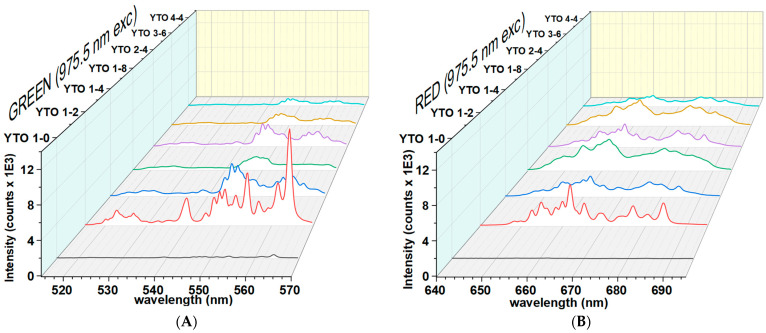
Visible upconversion spectra at illumination at 975.5 nm. (**A**): emissions from the ^4^H_11/2_,^4^S_3/2_→^4^I_15/2_ (green) transitions and (**B**): emissions from the ^4^F_9/2_→^4^I_15/2_ (red) transitions. It is seen, that in the case of 975.5 nm irradiation, the green emission intensities are comparable with the red ones, excepting YTO 1-2, which is a special case.

The brightest spots on the surfaces of the pellets were targeted with 150 mW illumination power (continuous wave) and the spectra were measured for two close wavelengths, 973.5 nm and 975.5 nm. Figure 8 and Figure 9 present the upconversion spectra for the visible part for both values of the excitation radiation wavelength.

The difference between these excitation energy values is about 20 cm^−1^, but the effects on the upconversion response are important. This fact is an indication that the upconversion mechanism can be linked to some detuning of some yet-unspecified resonant energy transfers between Er^3+^ and Yb^3+^ ions. Not only are the upconversion spectral compositions different for the two incident wavelengths, but also the emitted power curves are altered.

This high sensitivity with the incident radiation wavelength of Er,Yb:YTO ceramics is different from the other ceramics (Y_2_O_3_, BaY_2_ZnO_5_, BaGd_2_ZnO_5_, CaGd_2_ZnO_5_, BaY_2_O_4_, and BaY_2_MgO_5,_ for the same dopant concentration) that we tested. In the 973.5–975.5 nm excitation wavelength range, these ceramics display a similar upconversion spectra with a linear dependence of the emitted power vs. incident power, unlike Y_2_TiO_5_.

**Figure 10:**
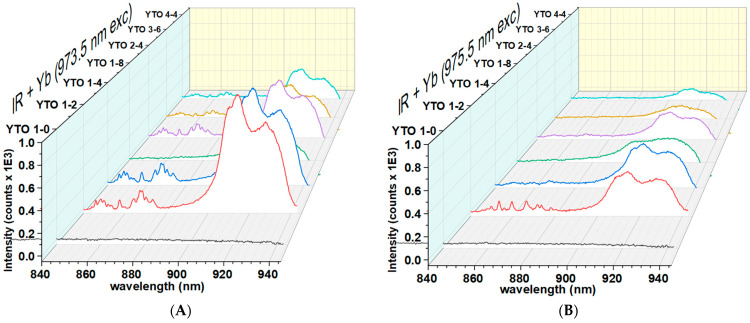
Er^3+^ emission at 865 nm (from 840 nm to 882 nm) corresponding to ^4^S_3/2_→^4^I_13/2_ transition and the anti-Stokes sideband (from 900 nm to 940 nm) of the emission of Yb^3+^ from the transition ^7^F_5/2_→^7^F_7/2_ (ground state of Yb^3+^). (**A**) Illumination at 973.5 nm and (**B**) illumination at 975.5 nm. The intensities are almost double for the 973.5 illumination and the cause of this effect remains to be determined.

Figure 10 presents the peaks (between 840 nm and 882 nm) of the emission of the transition ^4^S_3/2_→^4^I_13/2_ of Er^3+^ and the peaks between 900 nm and 940 nm, which are from the anti-Stokes sideband generated by the emission of Yb^3+^ when decaying from ^2^F_5/2_ to ^2^F_7/2_. Their widths and intensities are indications that the phononic interactions are significant. Also, they help to estimate the phonon energy for Y_2_TiO_5_ at about 610 cm^−1^.

It can be seen that, in the case when the excitation was performed at 973.5 nm radiation, the anti-Stokes peaks for Yb^3+^ are almost double those in the case with 975.5 nm. The YTO 1-8 case is an exception, revealing a diminished interaction between the phonons and Yb^3+^ ions.

One can observe in Figure 10 the variability in the ^4^S_3/2_→^4^I_13/2_ transition of Er^3+^ across the dopant concentrations and also in the wavelength of the exciting radiation. For the 975.5 nm excitation, it is observed that a higher Yb^3+^ concentration promotes the decay to the ground of Er^3+^, instead of being trapped to ^4^I_13/2_. At the 973.5 nm excitation, it is seen that Er^3+^ has a greater tendency to reach ^4^I_13/2_, only 8% of Yb^3+^ strongly hindering that. The mechanism by which the Yb^3+^ ions are influencing the Er^3+^ energy level transitions, when embedded in Y_2_TiO_5_ crystal matrix, will be the subject of further research.

## 4. Discussion

### 4.1. Fitting the Upconversion Spectra

**Figure 11:**
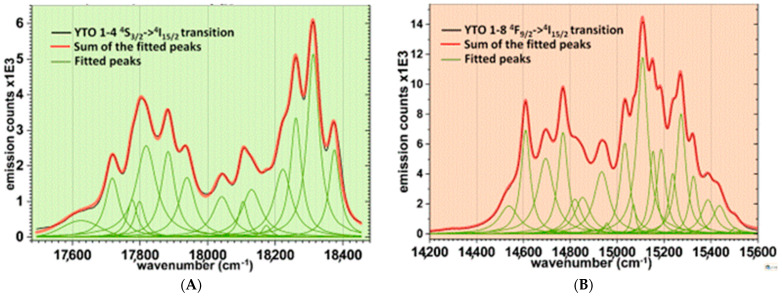
(**A**) The fitting of the Er^3+^ green emission (^4^S_3/2_→^4^I_15/2_) from the YTO 1-4 pellet and (**B**) the fitting of the Er^3+^ red emission (^4^F_9/2_→^4^I_15/2_) from the YTO 1-8 pellet, both at an illumination of 975.5 nm. The fitting is very good and the uniformity of the form factors of the peaks shows that the Stark levels of the involved manifolds have close lifetimes.

The spectra with the most intense peaks of Y_2_TiO_5_:Er,Yb ceramics were chosen for fitting. These were YTO 1-4 for the green part (^4^S_3/2_→^4^I_15/2_) and YTO 1-8 for the red part (^4^F_9/2_→^4^I_15/2_). The fitting squared error is under 10^−9^, and the results are shown in Figure 11. The fitted peaks data are presented in the Supplementary Material (Tables S2 and S3). The homogeneous broadened Lorentzian profiles and the relatively large FWHM are indications that the de-excitation of the Er^3+^ ions is influenced by phononic interactions. While the two Stark manifolds of ^4^S_3/2_ are easily detectable (separation at 18000 cm^−1^), the five ones in the case of ^4^F_9/2_ are a bit harder to separate.

### 4.2. Comments on the Upconversion Spectra

When only Er^3+^ is present, the UC is very weak, as can be seen in Figure 8 and Figure 9. The presence of Yb^3+^ ions greatly increases the efficiency due to the enhanced capture of the incident NIR radiation. The fitted peaks are Lorentzian, and the broadening of the lines is homogeneous, which indicates that the Er^3+^ ions are trapped in a crystalline phase. The high value of FWHM shows that the local field strength is lower than in the case of Y_2_O_3_, indicating that the Er^3+^ ions are being more loosely trapped when replacing Y^3+^ in the Y_2_TiO_5_ lattice.

Using the VESTA 3 software [32], the average Er^3+^↔O^2-^ distances, i.e., the average radii of coordination, were calculated, and they are 2.305 Å in the case of cubic Y_2_O_3_ (C_2v_ site) (VI coordination) and 2.363 Å in the case of orthorhombic Y_2_TiO_5_ (VII coordination). If this small difference (2.5%) is to account for the large differences between the average widths of the spectral lines in the case of Y_2_O_3_ (average FWHM ≈ 33 cm^−1^) [37] compared with those of Y_2_TiO_5_ (average FWHM ≈ 50 cm^−1^), it remains to be investigated. Also, this suggests that the smaller lifetimes of the excited levels in the case of the Er^3+^ ions trapped in Y_2_TiO_5_ may have other causes than the immediate sphere of coordination.

### 4.3. YTO 1-2 Special Case

**Figure 12:**
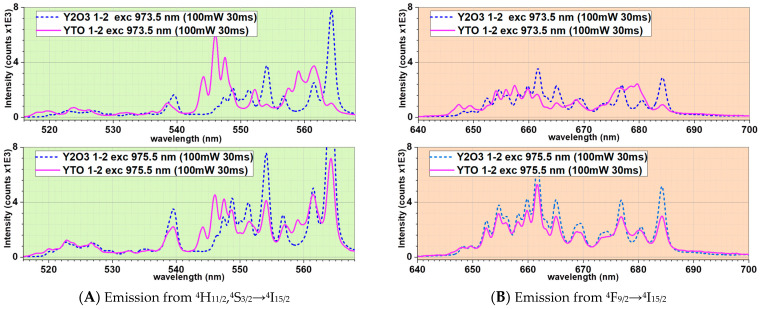
A comparison between the spectra for the emissions from the transitions (**A**) ^4^H_11/2_,^4^S_3/2_→^4^I_15/2_ and (**B**) ^4^F_9/2_→^4^I_15/2_ in the case of YTO 1-2 (continuous magenta lines) and Y_2_O_3_ 1-2 (dotted blue lines). The spectra for YTO are scaled by the appropriate factor to better compare with those of Y_2_O_3_. For both crystal hosts, the peak positions are the same, showing that the crystal field strength is the same. The average distance between Y^3+^↔O^2-^ is 2.3045 Å in Y_2_O_3_ and 2.3629 Å in Y_2_TiO_5_ and this 2.5% difference should have a visible effect in the widths of the splitting.

It is worth noting that the YTO 1-2 ceramic has peculiar behavior. The upconversion spectrum for the excitation at 975.5 nm has an almost identical shape to the Y_2_O_3_ doped with 1% Er^3+^ and 2% Yb^3+^ (Y_2_O_3_ 1-2) [37]. The position and intensity of the peaks are the same for the red emission, while for the green emission, minor differences are observed (Figure 12).

While the peak positions are the same, their intensity distributions show that, in the case of the 973.5 nm excitation, the upper level of the Stark-degenerated ^4^S_3/2_ is more populated. This is unexpected because the Stark splitting, caused by the intensity and shape of the embedding crystal field, should be different in the two cases (Y_2_TiO_5_ vs. Y_2_O_3_), with the coordination polyhedra of Er^3+^ and the Er^3+^–O^2-^ distances not being the same.

Moreover, while the upconversion spectrum of Y_2_O_3_ 1-2 has the same shape for both the incident IR wavelengths (Figure 12), for YTO 1-2, the spectra differ drastically from 973.5 nm to 975.5 nm, as can be seen in Figure 8 and Figure 9. As the energy difference is only 20 cm^−1^ between 973.5 nm and 975.5 nm, such behavior can only be due to other causes than the coordination polyhedra shape and dimension.

The only parameter that is common for both cases is the interionic Er^3+^↔Yb^3+^ average distance distributions, and the almost identical shape of the spectra in the 975.5 nm case is a strong indication that the upconversion mechanism has, at its roots, not only the general considered energy transfers between the Er^3+^ and Yb^3+^ ions, but also some interactions between the Er^3+^ ions and an overall photon field maintained by the Yb^3+^ ions, disregarding the peculiarities of the host matrix.

To the best of our knowledge, this behavior is not explained in the literature, its origin is unknown, and a segregation of some of the Y_2_O_3_ phase was suspected. The synthesis and analyses of YTO 1-2 were repeated three times, but each time the same results were found, with no Y_2_O_3_ phase shown in the XRD patterns, and the upconversion spectra being reproducible. The best indication that the Y_2_O_3_ phase is not segregated and cannot be the cause for the unusual spectrum is that, for the 973.5 nm case, the upconversion spectrum differs from that of the 975.5 nm case, as seen in Figure 8 and Figure 9 for YTO 1-2.

### 4.4. Comparison of the Upconversion Efficiency

**Figure 13:**
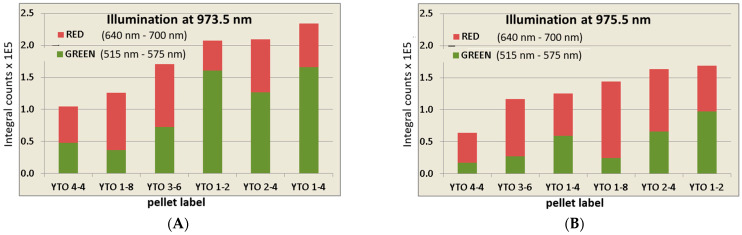
Integral emission intensities in the green domain (515 nm–575 nm) and in the red domain (640 nm–700 nm) for each dopant case ordered from total (red + green) lowest to highest for excitation radiation of (**A**) 973.5 nm and (**B**) 975.5 nm. Observe the sample order and how the increasing dopant concentrations do not necessarily improve the UC efficiency.

The integral intensities for the entire visible emission (in the red and green domains), for both the illumination wavelengths, are presented in Figure 13. The graphs are for an illumination with 150 mW for 30 ms. One can notice that YTO 4-4 has the lowest efficiency in all cases, i.e., Er^3+^ quenches itself. YTO 1-4, which is the brightest at 973.5 nm, is less responsive to 975.5 nm. In the 973.5 nm case, the green emission is brighter and quickly becomes hindered at 975.5 nm. For an energy difference of only 20 cm^−1^ between 973.5 and 975.5 nm, this is unexpected.

**Figure 14:**
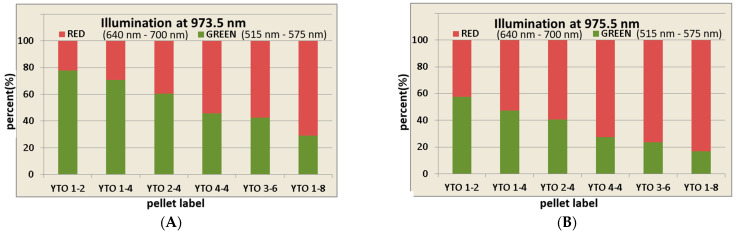
The percentage of green (515 nm–575 nm) emission vs. red (640 nm–700 nm) emission in the total red and green domain for an excitation radiation of (**A**) 973.5 nm and (**B**) 975.5 nm. An increasing Yb^3+^ concentration promotes the ^4^F_9/2_ level of Er^3+^, increasing the Er^3+^ concentration produces the same effect indicating that the transition from ^4^S_3/2_ →^4^F_9/2_ is governed rather by the dopant interionic distances than by the energy transfers with the lattice.

In Figure 14, the samples are ordered by decreasing the percentage of green in the total emission, clearly showing the influence of Yb^3+^ in shifting the population of the excited Er^3+^ ions toward ^4^F_9/2_. It is remarkable is that the excitation at 973.5 nm induces brighter green than red emissions. However, the upconversion efficiency for the Y_2_TiO_5_ matrix is lower than that of Y_2_O_3_ [37].

From Figure 13 and Figure 14, one can observe that increasing the Yb^3+^ concentration (YTO 1-2, 1-4, 1-8) facilitates the transition from ^4^F_9/2_→^4^I_15/2_, whereas maintaining the Yb^3+^ content and increasing the Er^3+^ concentration (YTO 1-4, 2-4, 4-4) maintains the red intensity while the total emission is quenched because of the decreasing of the Er^3+^↔Er^3+^ distances.

### 4.5. Correlation with the Er^3+^↔Er^3+^ and Er^3+^↔Yb^3+^ Interionic Distances

**Figure 15:**
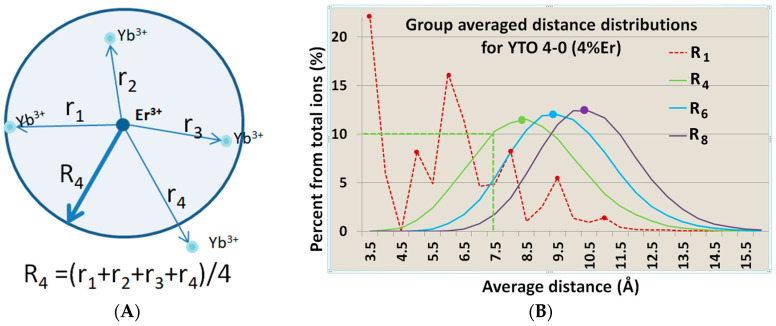
(**A**) A 2D example of how the average distance R_4_ from a random Er^3+^ ion to the closest four (randomly distributed) Yb^3+^ (or Er^3+^) neighbors was calculated. (**B**) Example of the distributions of the average interionic distances from Er^3+^↔Er^3+^ when the total Er^3+^ concentration is 4%. The curves are for the 1, 4, 6, and 8 closest Er^3+^ neighbors. As expected, the distributions are Poissonian. The vertical axis represents the percentage of Er^3+^ ions that have the respective number of neighbors at the average distance specified on the horizontal axis, e.g., 10% of the Er^3+^ ions have, for the closest four neighbors, an average distance of 7.5 Å (green curve and green dotted straight lines), and 24% of Er^3+^ ions have the closest single Er^3+^ neighbor at 3.5 Å (red dotted graph, left-up red point).

Such a behavior must be correlated with the distances between the dopants, either Er^3+^ ↔Er^3+^ or Er^3+^↔Yb^3+^. This was performed using a numerical simulation of randomly placed Er^3+^ and Yb^3+^ dopant ions (substituting Y^3+^ ions) in a cubic lattice (500 Å edge) of crystalline orthorhombic Y_2_TiO_5_ and computing the distributions of the average distances from the *N* = 4, 6, 8, closest Yb^3+^ neighbors of each Er^3+^ ion to itself, as exemplified in Figure 15A. The examples for the Er^3+^↔Er^3+^ distance distributions in the case of 4% Er^3+^ are shown in Figure 15B and Table 3.

**Figure 16:**
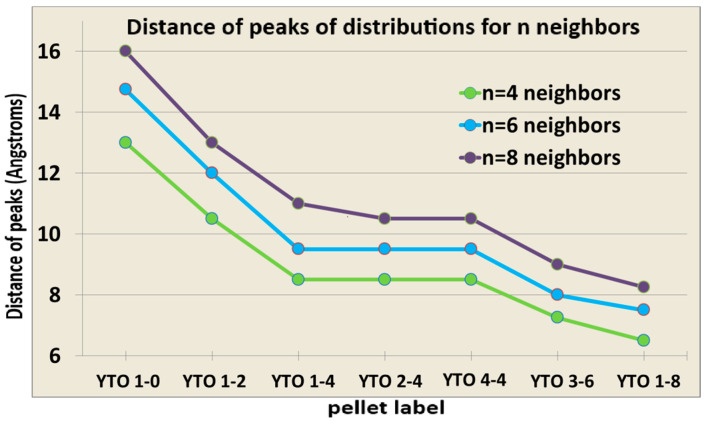
Graph with data from Table 3 with the average distances (vertical axis), in Å, from a random Er^3+^ to the closest 4, 6, and 8 Yb^3+^ neighbors (or Er^3+^ in the case of YTO 1-0). For cases YTO 1-4, YTO 2-4, and YTO 4-4, the distributions for the Er^3+^↔Yb^3+^ distances are the same; only the Er^3+^↔Er^3+^ average distances are decreasing. (Same colors as in Figure 15B).

Figure 16 presents the samples, ordered by the decrease of the average Er^3+^↔Yb^3+^ distances, and it can be seen that, in the case of YTO 1-2, 2-4, and 4-4, the average distances from the activators to the sensitizers are almost the same. Nevertheless, the variability of the spectral shape and compositions for the UC responses in the respective cases is a clear indication that the Er^3+^↔Er^3+^ interaction must be taken into consideration when designing for UC efficiency, because increasing the Er^3+^ content does not necessarily lead to better performance.

**Figure 17:**
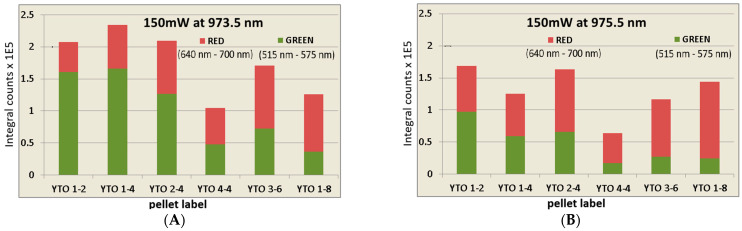
Total visible spectrum (red and green) integral counts for each case of illumination, (**A**) 973.5 nm and (**B**) 975.5 nm, ordered according to the average Er^3+^↔Yb^3+^ neighboring radii R_4_, R_6_, and R_8_, from Figure 16, for the two cases of the excitation wavelengths. Observe how YTO 4-4 has the lowest UC efficiency in both cases.

Figure 17 shows the total intensities for the two cases (973.5 nm and 975.5 nm) with the samples ordered by the decrease in the average radii (any of R_4_, R6, and R_8_), according to the graph in Figure 16. The drop in intensities for the YTO 4-4 case should be noted, clearly showing Er^3+^↔Er^3+^ quenching.

### 4.6. Emitted Power Variation Curves

**Figure 18:**
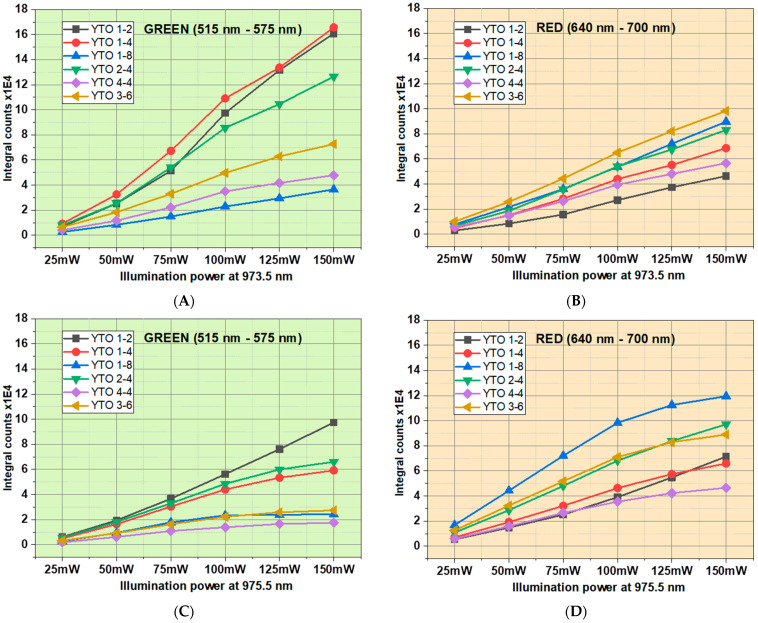
(**A**,**C**) The integral counts for the green (515 nm–575 nm) emission and (**B**,**D**) red (640 nm–700 nm) emission parts of the upconversion spectra. Both cases of illuminating wavelengths are shown. Observe the saturation starting at 100 mW in the case of 975.5 nm.

The power of the incident IR laser radiation was increased from 25 mW to 150 mW, by steps of 25 mW. The same spot was illuminated and the UC spectra were measured for both wavelengths, 973.5 nm and 975.5 nm. The photoluminescent integral intensities, both for the green (^4^H_11/2_,^4^S_3/2_→^4^I_15/2_) and red (^4^F_9/2_→^4^I_15/2_) emissions, are displayed in Figure 18.

It is interesting to observe that, in the case of 975.5 nm, the output power starts to saturate beyond 100 mW, while in the case of 973.5 nm, the linearity is preserved. This shows that, at a certain threshold of local power density for 975.5 nm, a certain percentage of Er^3+^ ions involved in the UC process either prefer other decay channels than directly to the ground state, or the emitted photons are absorbed (notable for the YTO 1-8 and YTO 3–6 samples).

Also, there is a strong variability in the concentrations of the doping ions for the output power for the two incident wavelengths. Notable is the case of YTO 1-4, where the output power for green emission at 973.5 nm is much higher than that of red emission (bright red (round dots) line in Figure 18A,B), while for YTO 1-8, the green emission is strongly suppressed (blue line (up triangles) in Figure 18C,D).

## 5. Conclusions

Erbium and ytterbium-doped diyttrium titanate ceramic samples were obtained at 1250 °C from oxide powders, which were synthesized using the sol–gel method. The characterization using XRD and SEM showed that the doped Y_2_TiO_5_ ceramics’ microstructure and phase composition were very sensitive to the dopant concentrations. When the Yb^3+^ concentration is increased, additional hexagonal and fluorite phases appear, which fixes the maximum threshold for the Yb^3+^ concentration at 10%, beyond which the Y_2_TiO_5_ orthorhombic phase becomes heavily distorted.

The samples were illuminated with 973.5 and 975.5 nm from a thermally tuned laser diode, and the resulting UC visible spectra were measured and characterized. In the case of Y_2_TiO_5_:1%Er^3+^:2%Yb^3+^, we observed that the upper Stark level of ^4^S_3/2_ of Er^3+^ becomes more populated when illuminated with 973.5 vs. 975.5 nm, this fact inducing a notable change in the hue and peak intensities in the UC response. Also, the emitted green–red intensities, both relative and absolute, for the other samples were observed and compared for the 973.5 and 975.5 nm irradiations, showing notable differences that were not observed in other crystalline lattices that we studied or found in the literature. The curves relating the emitted intensity versus the excitation power of the incident radiation were not linear, with a saturation tendency beyond 100 mW for green (515–575 nm), in the case of 973.5 nm irradiation, and for red (640–700 nm), in the case of 975.5 nm illumination.

Simulations were performed for the distributions of the interionic distances for Er^3+^↔Er^3+^ and Er^3+^↔Yb^3+^, and the samples were ordered by the distance of the maximum for each case of the dopant concentrations. It is seen that Er^3+^↔Er^3+^ quenching takes place when Er^3+^ is 4%, regardless of the Yb^3+^ concentration, showing that Er^3+^↔Er^3+^ energy transfers are more prevalent than those between Er^3+^ and Yb^3+^. The fact that the upper Stark level of ^4^S_3/2_ is more populated when the illumination is performed with 973.5 nm vs. 975.5 nm is puzzling, since the distance between the two levels of ^4^S_3/2_, split by the crystal field of Y_2_TiO_5_, is more than 20 cm^−1^, which is the energy difference between 973.5 nm and 975.5 nm.

The source of this energy difference will be the subject of further investigations; nevertheless, the sensitivity of the effect calls for practical engineering applications with a high degree of precision. Some of these are laser diode wavelength tuning, cheap laser diode quality control, laser wavelength indicators, crystalline structural modification indicators when YTO is incorporated in other bulk ceramics, cheap doppler sensors with under 2 nm precision detection for non-inertial optical fiber gyroscopes, and last but not least, forgery prevention by including certain YTO nanodots in the protected samples.

## Figures and Tables

**Table 1:** Percentages of Er^3+^ and Yb^3+^ dopant ions replacing Y^3+^ ions in Y_2_TiO_5_ and the label of the samples. The Er/Yb concentration ratios are chosen to be either powers of 2 or ratios of 2.

Dopant Concentration (%)		Sample Label
Er^3+^	Yb^3+^	
0	0	YTO 0-0
0	1	YTO 0-1
1	0	YTO 1-0
1	2	YTO 1-2
1	4	YTO 1-4
1	8	YTO 1-8
2	4	YTO 2-4
4	4	YTO 4-4
3	6	YTO 3-6

**Table 2:** Ionic and crystal radii for Y^3+^, Er^3+^, and Yb^3^.

Ion in VII Coordination	Crystal Radius (Å)	Ionic Radius (Å)
Y^3+^	1.100	0.960
Er^3+^	1.085	0.945
Yb^3+^	1.065	0.925

**Table 3:** Peaks of the average distance distributions for the different percentile Er^3+^ and Yb^3+^ concentrations from a random Er^3+^ ion to the first group of 4, 6, and 8 neighbors, be they Er^3+^ (for 1-0, 2-0, 4-0) or Yb^3+^, as specified in the first column. The last line in the table shows the peak values for YTO 4-0, whose colored distributions and peaks are shown in Figure 15B (green; blue and magenta lines).

Ceramic %Er-%Yb	*N* = 4 Neighbors Peak at (Å)	*N* = 6 Neighbors Peak at (Å)	*N* = 8 Neighbors Peak at (Å)
YTO 1-8	6.5	7.5	8.3
YTO 3-6	7.3	8.0	9.0
YTO 4-4	8.5	9.5	10.5
YTO 2-4	8.5	9.5	10.5
YTO 1-4	8.5	9.5	11.0
YTO 1-2	10.5	12.0	13.0
YTO 1-0	13.0	14.8	16.0
YTO 2-0	10.5	12.0	13.0
**YTO 4-0**	**8.5**	**9.5**	**10.3**

## Data Availability

The raw data of this paper will be made available by authors on reasonable request.

## Supplementary Materials

The following supporting information can be downloaded at: https://www.mdpi.com/article/10.3390/ma17163994/s1. Figure S1. The measurement setup for the upconversion spectra; Figure S2. X-ray diffractograms for YTO 2-4, YTO 4-4, and YTO 3-6 ceramic samples; Figure S3. The surfaces of the pellets after polishing. The illumination is frontal from the LED lights of the microscope. Observe the uniformity of the size of the microcrystals for each dopant case showing that the thermal field during sintering was uniform. The variation of the average size of the microcrystals show how the dopant concentrations influence the crystallization process; Figure S4. Polyhedral view of the unit cell of the orthorhombic crystal structure of Y_2_TiO_5_. Y^3+^ coordination polyhedra with green-gray, Ti^4+^ coordination polyhedra with light blue; Figure S5. SEM images (magnification x2000, field of view 72 μm) for powders obtained at 1250 °C (left images) and ceramic pellets (right images). Observe the morphology of the particles, which resemble glassy shards with a good similarity to volcanic ash; Figure S6. Images of the pellets at small angle (from right) IR laser illumination. The IR laser wavelengths are 973.5 nm for left images and 975.5 nm for the right ones. Observe the spots and the mosaics. The stripe variation in brightness is due to the variability of the laser field emitted by the diode. For the YTO 1-0 case the upconversion emission is weaker than the reflected residual IR light which passes through the IR filter of the camera; Figure S7. EDX analysis of the pellets (A) YTO 1-4 and (B) YTO 1-8; Figure S8. IR response to the illumination with IR laser of the reference pellet YTO 0-1 (only Yb^3+^); Table S1. The phases content of the ceramic samples; Table S2. The peak data resulted from the fitting of the green spectrum; Table S3. The peak data resulted from the fitting of the red spectrum.
