# Supplementary material for: Nonlinearity of the Upconversion Response of Er3+ in Y2TiO5:Er3+,Yb3+ Ceramics When Varying the Wavelength of Incident NIR Excitation Radiation
Source: Materials (Basel). 2024 Aug 11;17(16):3994. doi: 10.3390/ma17163994 (PMC11356084; doi:10.3390/ma17163994)
Supplement: Supplementary file 1 [file materials-17-03994-s001.zip › materials-3148632-supplementary.pdf]

Nonlinearity of the upconversion response of Er^{3+} in $Y_2TiO_5:Er^{3+},Yb^{3+}$ ceramics when varying the wavelength of incident NIR excitation radiation

Liviu Dudaș[1], Daniela Berger[1] and Cristian Matei[1]

1 National University for Science and Technology POLITEHNICA of Bucharest, Romania; liviu_dudas@yahoo.com (L.D.), daniela.berger@upb.ro (D.B.), cristian.matei@upb.ro (C.M.)

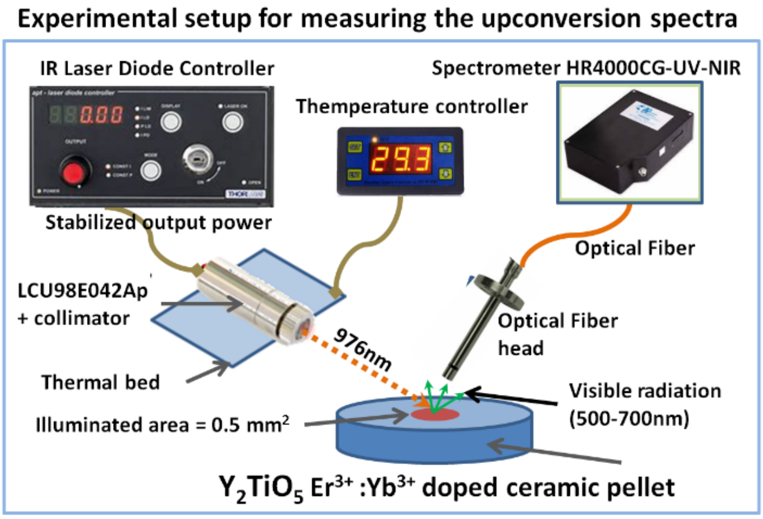

Figure S1. The measurement setup for the upconversion spectra

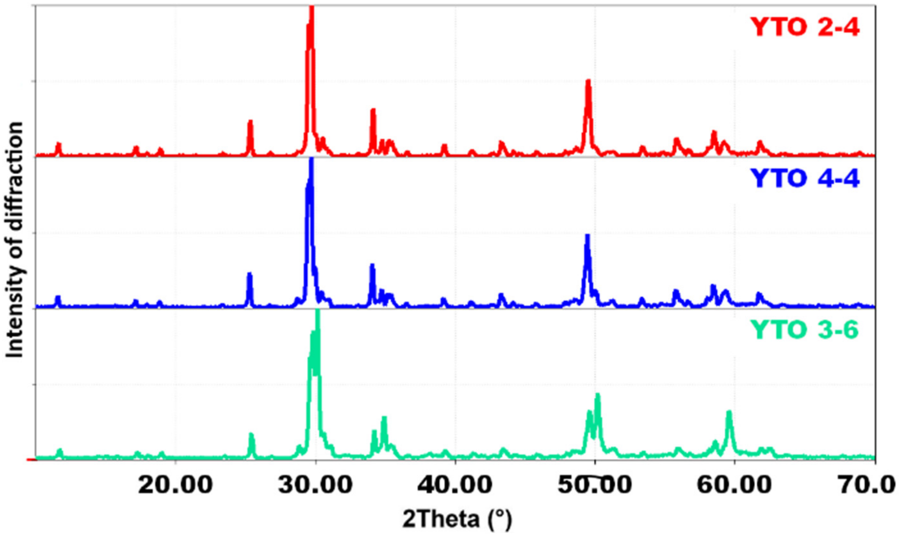

Figure S2. X-ray diffractograms for YTO 2-4, YTO 4-4 and YTO 3-6 ceramic samples.

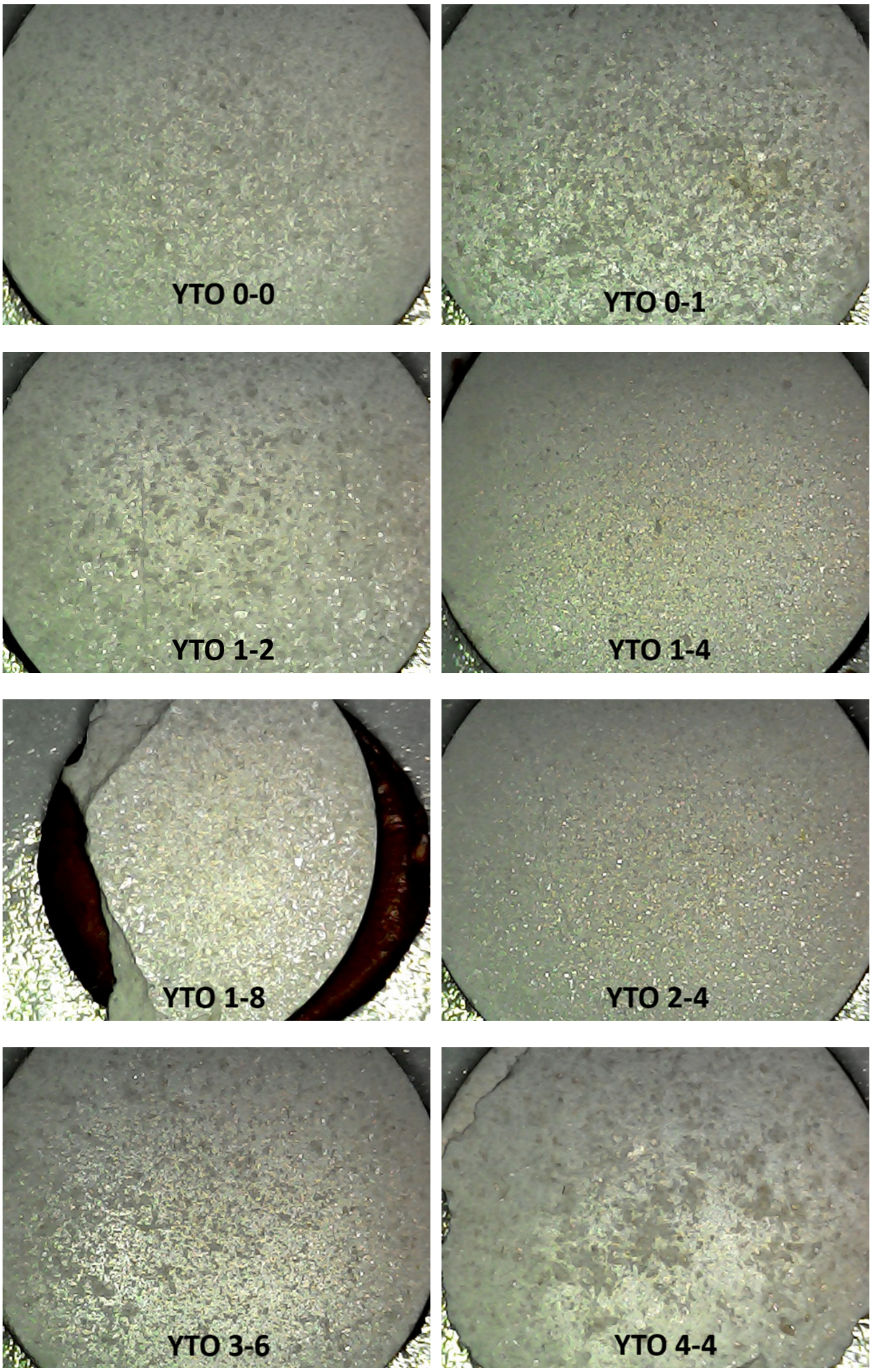

Figure S3. The surfaces of the pellets after polishing. The illumination is frontal from the LED lights of the microscope. Observe the uniformity of the size of the microcrystals for each dopant case showing that the thermal field during sintering was uniform. The variation of the average size of the microcrystals show how the dopant concentrations influence the crystallization process.

Table S1. The phases composition of the ceramic samples.

Ceramic pellet composition	percent	[ICDD card]	phase type	formula
YTO 0-0	21.1%	[01-076-0787]	orthorhombic	Y_2TiO_5
	48.9%	[00-040-0795]	orthorhombic	Y_2TiO_5
	30.00%	[00-024-1426]	orthorhombic	Y_2TiO_5
YTO 0-1	47.6%	[01-076-0787]	orthorhombic	Y_2TiO_5
	44.3%	[00-040-0795]	orthorhombic	Y_2TiO_5
	8.1%	[00-024-1426]	orthorhombic	Y_2TiO_5
YTO 1-0	36.3%	[01-076-0787]	orthorhombic	Y_2TiO_5
	42.3%	[00-040-0795]	orthorhombic	Y_2TiO_5
	21.3%	[00-024-1426]	orthorhombic	Y_2TiO_5
YTO 1-2	35.8%	[01-076-0787]	orthorhombic	Y_2TiO_5
	44.3%	[00-040-0795]	orthorhombic	Y_2TiO_5
	19.9%	[00-024-1426]	orthorhombic	Y_2TiO_5
YTO 1-4	35.1%	[01-076-0787]	orthorhombic	Y_2TiO_5
	46.4%	[00-040-0795]	orthorhombic	Y_2TiO_5
	18.5%	[00-024-1426]	orthorhombic	Y_2TiO_5
YTO 1-8	27.8%	[simulated]	fluorite	$Y_2Ti_2O_7$
	4.8%	[01-085-0001]*	orthorhombic	$YTiO_{.085}$
	67.5%	[00-027-0981]	hexagonal	Y_2TiO_5
YTO 2-4	7.3%	[01-076-0787]	orthorhombic	Y_2TiO_5
	67.5%	[00-040-0795]	orthorhombic	Y_2TiO_5
	25.2%	[00-024-1426]	orthorhombic	Y_2TiO_5
YTO 4-4	35.1%	[01-076-0787]	orthorhombic	Y_2TiO_5
	46.1%	[00-040-0795]	orthorhombic	Y_2TiO_5
	18.8%	[00-024-1426]	orthorhombic	Y_2TiO_5
YTO 3-6	33.1%	[simulated]**	fluorite	$Y_2Ti_2O_7$
	15.8%	[01-076-0787]	orthorhombic	Y_2TiO_5
	51.0%	[00-040-0795]	orthorhombic	Y_2TiO_5

N.B. the percent of ICDD card phases that, in the respective percentile combinations, matched best with the measured XR diffractograms for each ceramic pellet.

*ICDD card [01-085-0001] is for the formula ($YTiO_{2.085}$), the rest are for Y_2TiO_5,

**for the fluorite, the simulated XRD with unit cell edges of 5.15Å was used.

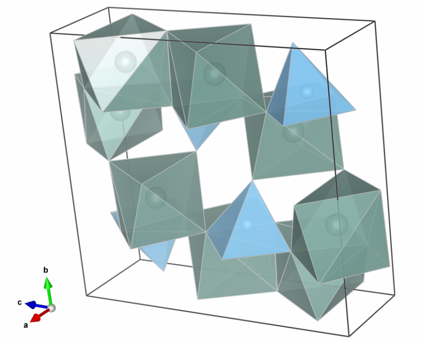

Figure S4. Polyhedral view of the unit cell of the orthorhombic crystal structure of Y_2TiO_5. Y^{3+} coordination polyhedra with green-gray, Ti^{4+} coordination polyhedra with light blue

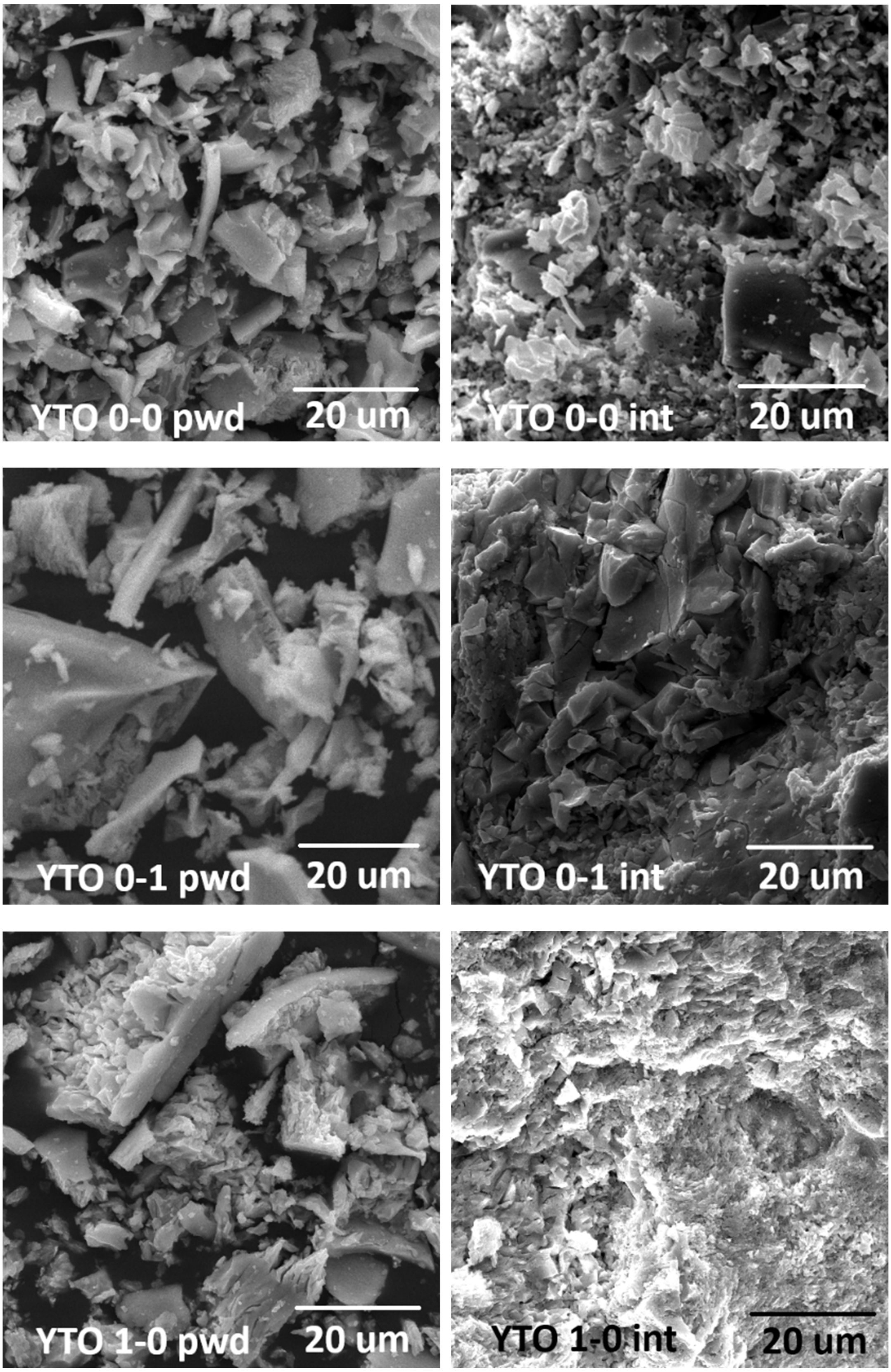
YTO 0-0 pwd
20 um
YTO 0-0 int
20 um
YTO 0-1 pwd
20 um
YTO 0-1 int
20 um
YTO 1-0 pwd
20 um
YTO 1-0 int
20 um

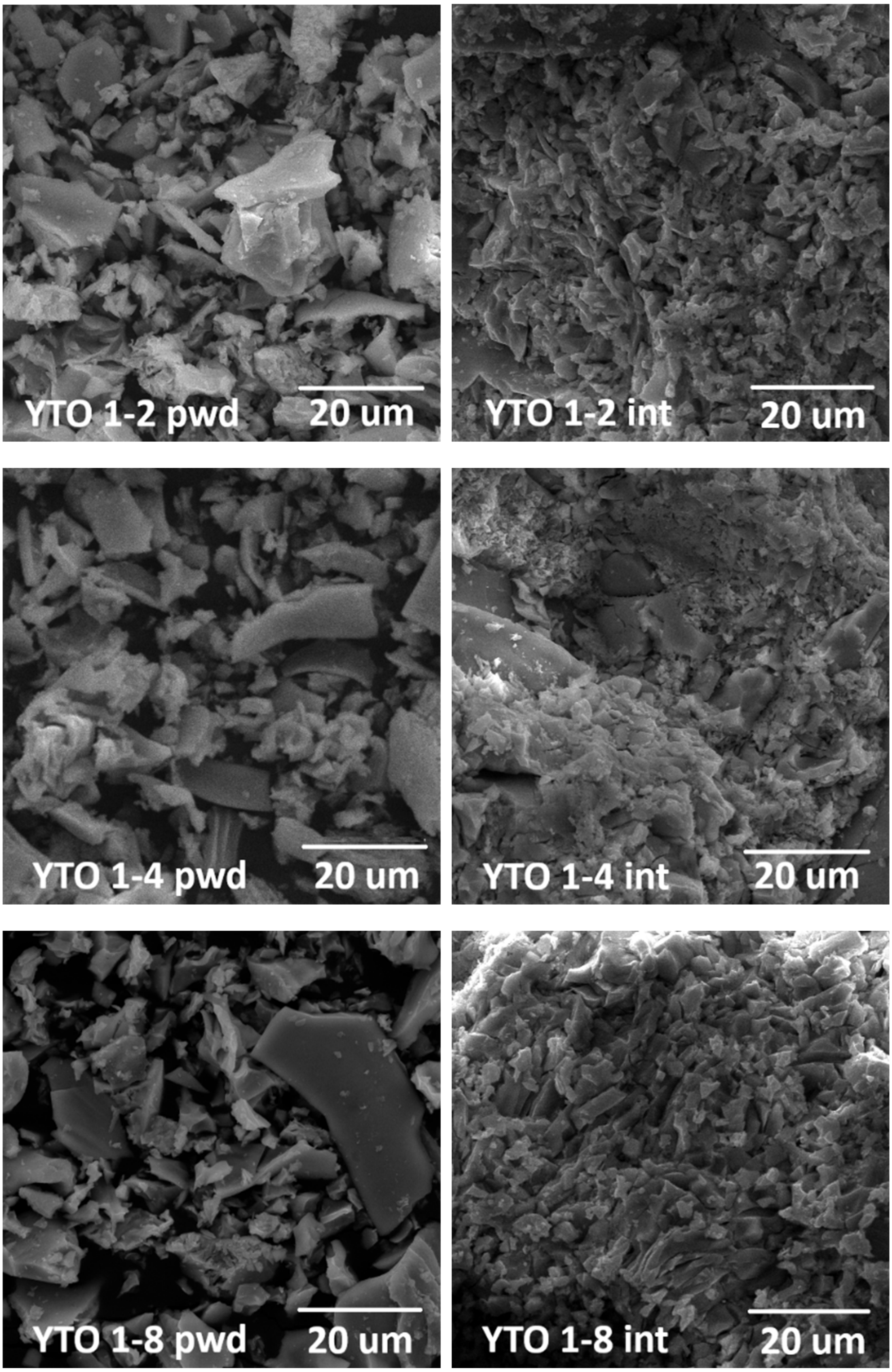
YTO 1-2 pwd
20 um
YTO 1-2 int
20 um
YTO 1-4 pwd
20 um
YTO 1-4 int
20 um
YTO 1-8 pwd
20 um
YTO 1-8 int
20 um

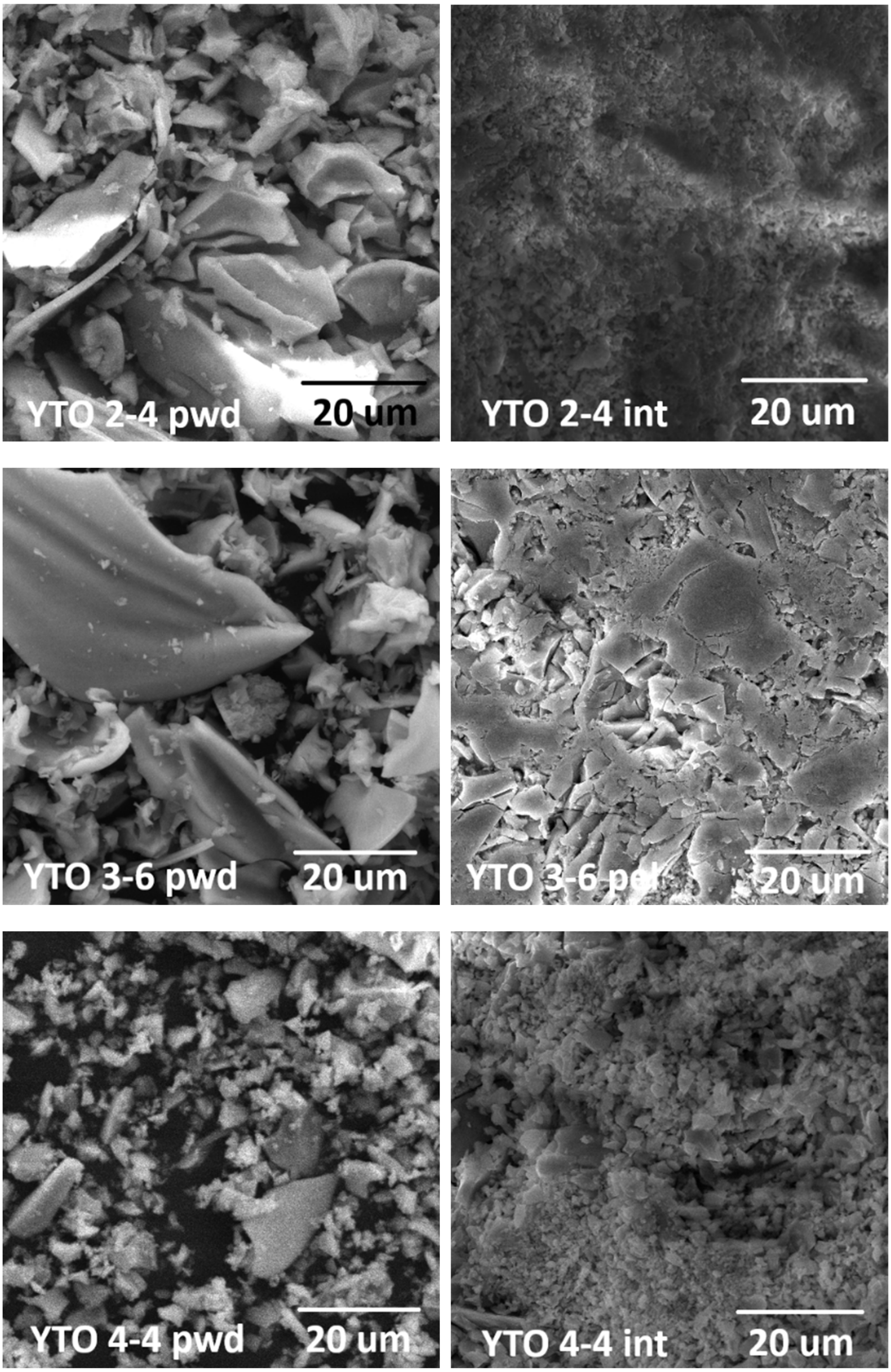

Figure S5. SEM images (magnification x2000, field of view 72 μm) for powders obtained at 1250 °C (left images) and ceramic pellets (right images). Observe the morphology of the particles, which resemble glassy shards with a good similarity to volcanic ash.

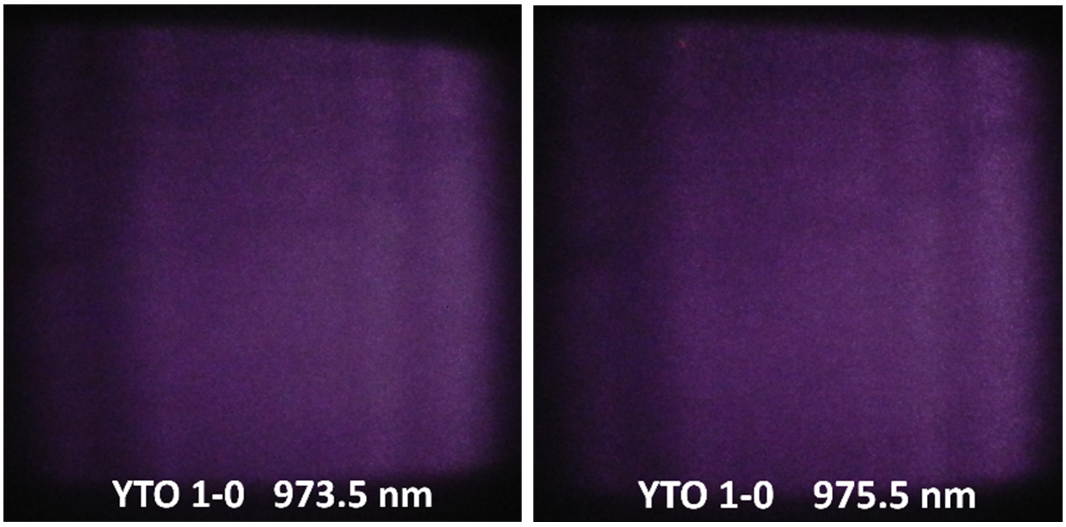

For YTO 1-0 the pictures show only the residual infrared passing the camera IR filter, the visible emission being too weak.

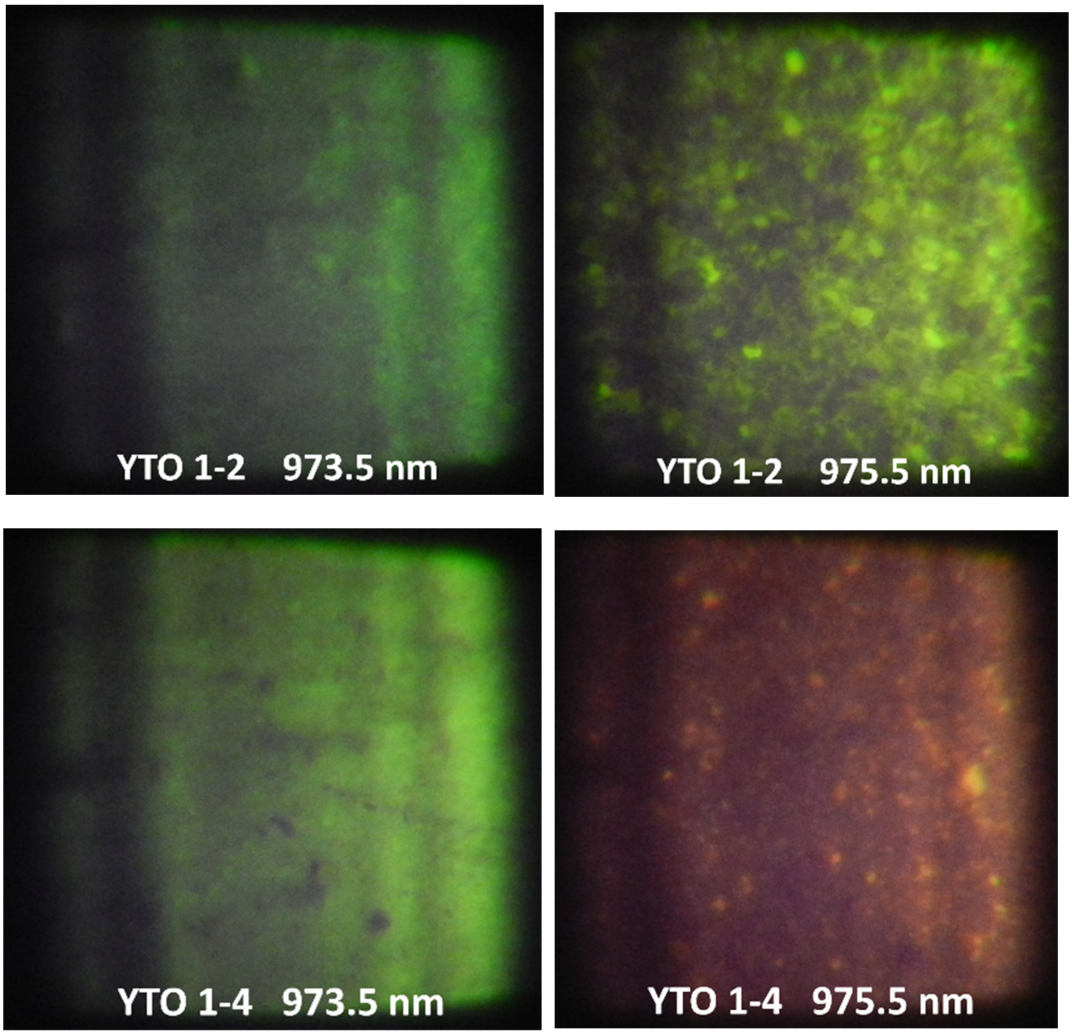

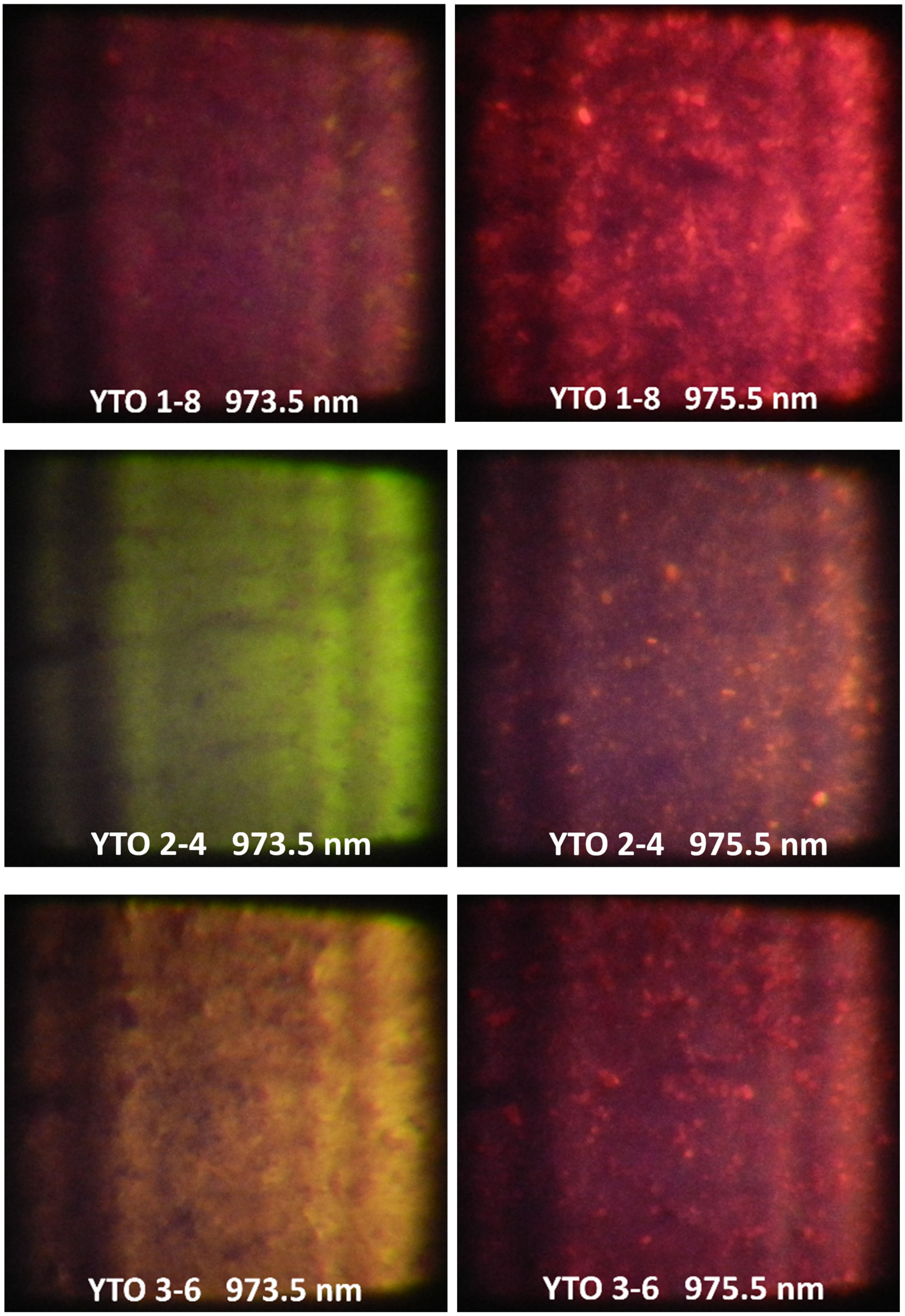
YTO 1-8 973.5 nm
YTO 1-8 975.5 nm
YTO 2-4 973.5 nm
YTO 2-4 975.5 nm
YTO 3-6 973.5 nm
YTO 3-6 975.5 nm

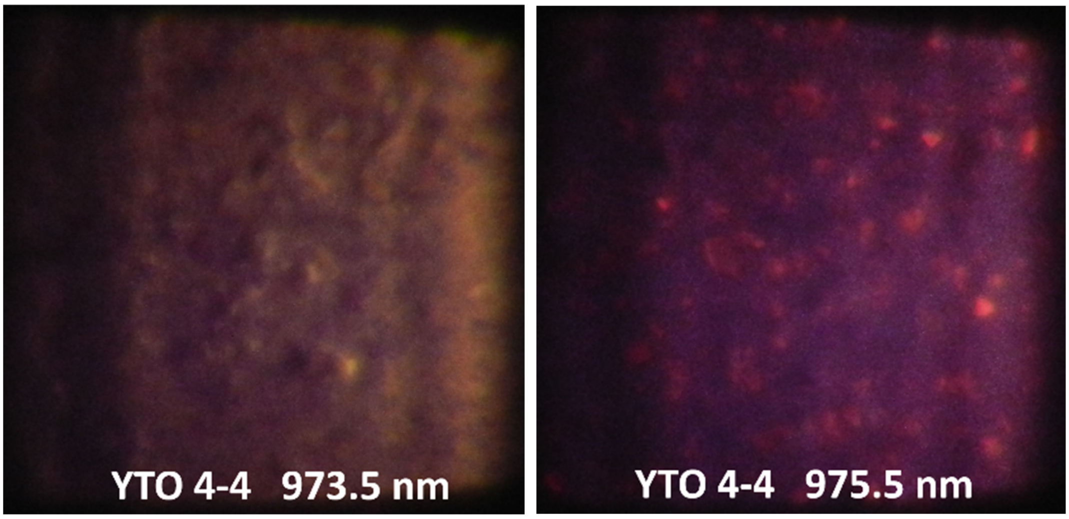

Figure S6. Imagines of the pellets at small angle (from right) IR laser illumination. The IR laser wavelengths are 973.5 nm for left images and 975.5 nm for the right ones. Observe the spots and the mosaics. The stripe variation in brightness is due to the variability of the laser field emitted by the diode. For the YTO 1-0 case the upconversion emission is weaker than the reflected residual IR light which passes through the IR filter of the camera.

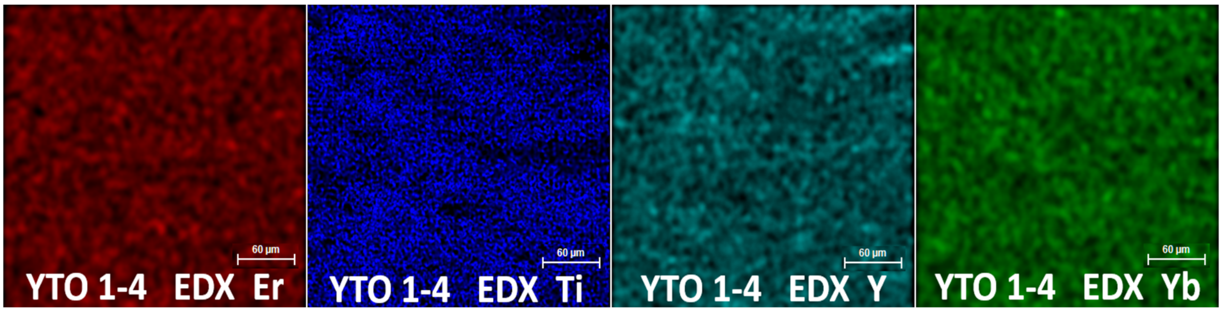

(A)

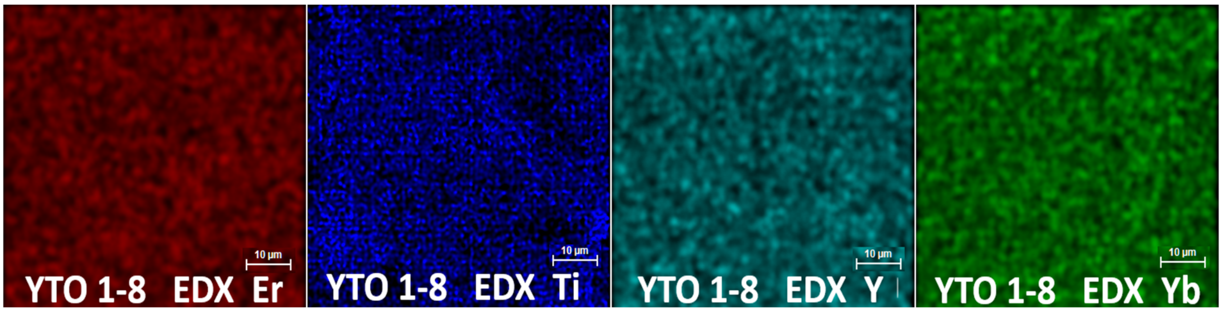

(B)

Figure S7. EDX analysis of the pellets (A) YTO 1-4 and (B) YTO 1-8

Nonlinearity of the upconversion response of Er^{3+} in $Y_2TiO_5:Er^{3+},Yb^{3+}$ ceramics when varying the wavelength of incident NIR excitation radiation

Table S1. The peak data resulted from the fitting of the green spectrum.

Peak Index	Peak Type	FWHM (cm^{-1})	Max Height	Center (cm^{-1})	Area IntgP
1	Gaussian	130.0	462.5	17626.0	3.1
2	Lorentz	50.0	1655.2	17718.4	6.0
3	Lorentz	35.9	1042.2	17777.1	2.8
4	Lorentz	34.9	1002.1	17798.9	2.6
5	Lorentz	72.0	2564.2	17818.3	13.4
6	Lorentz	49.0	2400.4	17883.2	8.7
7	Lorentz	57.0	1675.0	17938.9	7.0
9	Lorentz	65.0	1134.7	18042.0	5.4
10	Lorentz	32.0	1003.0	18104.8	2.4
11	Lorentz	68.9	1334.2	18130.1	6.7
12	Lorentz	29.0	329.3	18174.0	0.7
13	Lorentz	61.9	1906.3	18222.5	8.5
14	Lorentz	40.0	3342.3	18261.1	9.8
15	Lorentz	43.5	5136.9	18312.5	16.1
16	Lorentz	39.0	2448.5	18375.0	6.7

Table S2. The peak data resulted from the fitting of the red spectrum.

Peak Index	Peak Type	FWHM (cm^{-1})	Max Height	Center (cm^{-1})	Area IntgP
1	Gaussian	70.9	75.1	14285.0	0.1
2	Lorentz	100.0	1865.2	14537.7	4.1
3	Lorentz	47.6	6933.7	14609.3	7.5
4	Lorentz	82.3	5043.3	14695.1	9.3
5	Lorentz	50.2	6774.8	14769.1	7.7
6	Lorentz	70.3	2318.6	14819.7	3.7
7	Lorentz	79.2	2440.4	14852.5	4.3
8	Lorentz	78.7	4160.4	14934.3	7.4
9	Lorentz	29.2	737.1	14955.9	0.5
10	Lorentz	49.0	6059.5	15032.9	6.7
11	Lorentz	24.2	1963.4	15069.5	1.1
12	Lorentz	55.0	11788.1	15108.5	14.7
13	Lorentz	35.7	5529.9	15153.1	4.5
14	Lorentz	45.1	5656.9	15187.9	5.8
15	Lorentz	40.3	4027.6	15237.4	3.7
16	Lorentz	47.7	8016.4	15272.7	8.6
17	Lorentz	42.2	3853.2	15325.8	3.7
18	Lorentz	68.2	2280.1	15387.7	3.4
19	Lorentz	68.2	1890.0	15436.6	2.8
20	Lorentz	35.1	416.9	15503.6	0.3

Nonlinearity of the upconversion response of Er^{3+} in $Y_2TiO_5:Er^{3+},Yb^{3+}$ ceramics when varying the wavelength of incident NIR excitation radiation

In figure S8 are shown the peaks at 914 nm and 928 nm which corresponds to the anti-Stokes sidebands of Yb^{3+} when decaying from $^2F_{5/2}$ to the ground state ($^2F_{7/2}$). These peaks indicate that, for YTO matrix, an estimative optical phonon energy is about 610 cm^{-1}.

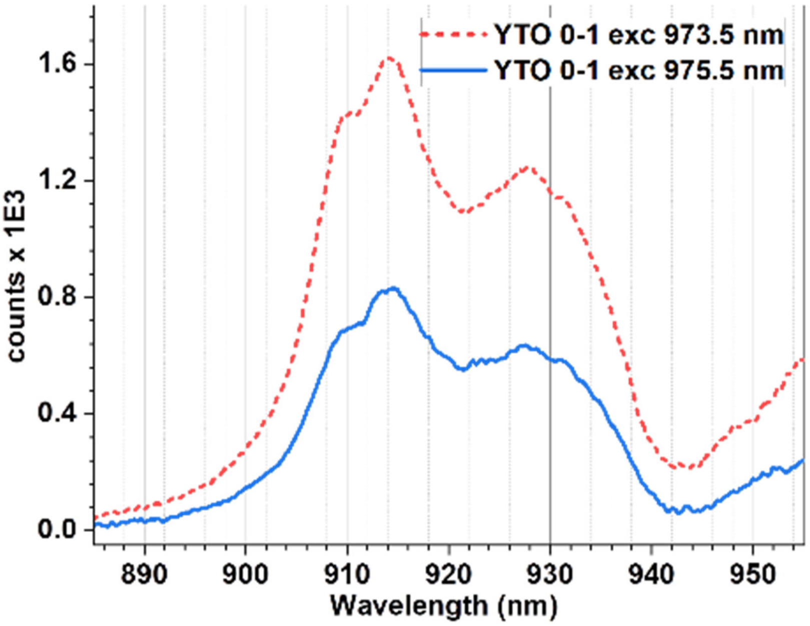

Figure S8. IR response to the illumination with IR laser of the reference pellet YTO 0-1 (only Yb^{3+}).
